# Extracellular vesicles produced by a large-scale protocol are therapeutically effective in preclinical model of Parkinson’s disease

**DOI:** 10.1093/stcltm/szag024

**Published:** 2026-04-29

**Authors:** Agnė Pociūtė, Virginijus Tunaitis, Arūnas Žebrauskas, Vladimirs Pilipenko, Baiba Jansone, Karina Narbute, Marianne Pultar, Matthias Hackl, Augustas Pivoriūnas

**Affiliations:** Department of Stem Cell Biology, State Research Institute Centre for Innovative Medicine, Vilnius, LT-01102, Lithuania; Department of Stem Cell Biology, State Research Institute Centre for Innovative Medicine, Vilnius, LT-01102, Lithuania; Department of Stem Cell Biology, State Research Institute Centre for Innovative Medicine, Vilnius, LT-01102, Lithuania; Faculty of Medicine and Life Sciences, University of Latvia, Riga, LV-1004, Latvia; Faculty of Medicine and Life Sciences, University of Latvia, Riga, LV-1004, Latvia; Latvian Biomedical Research and Study Center, Riga, LV-1067, Latvia; TAmiRNA GmbH, Vienna, 1110, Austria; TAmiRNA GmbH, Vienna, 1110, Austria; Department of Stem Cell Biology, State Research Institute Centre for Innovative Medicine, Vilnius, LT-01102, Lithuania

**Keywords:** extracellular vesicles, Parkinson’s disease, neuroprotection, scaling up production of extracellular vesicles, molecular fingerprinting of extracellular vesicles

## Abstract

**Background: **Therapeutic applications require large amounts of extracellular vesicles (EVs) that cannot be obtained by standard laboratory protocols. Since culturing parameters and isolation methods can significantly affect the molecular composition and therapeutic efficacy of EVs, the development of a new scale-up protocol should be followed by the molecular fingerprinting and validation of therapeutic potential *in vivo*.

**Methods: **We developed a new scale-up protocol based on microcarrier culture (3D) of immortalized human dental pulp stem cells in a spinning bioreactor and subsequent isolation of EVs by 2-step tangential flow filtration (TFF) and size exclusion chromatography (SEC).

**Results:** A new scale-up protocol increased EV yields by 463-fold. When compared with ultracentrifugation (UC), isolation using TFF/SEC substantially reduced the complexity of proteomic cargo, whereas culture conditions (2D vs. 3D) affected miRNA, but not mRNA and proteomic content of the EVs. We next compared the therapeutic efficacy of both EV products in 6-hydroxydopamine rat model of Parkinson’s disease (PD). The same amounts of EVs derived from standard 2D cultures by UC and a new large-scale protocol were intranasally administered to PD rats, where they similarly improved gait and cognitive functions, preserved nigrostriatal tyrosine hydroxylase density and suppressed neuroinflammation. Notably, both EV preparations were enriched in proteins and miRNAs associated with anti-oxidative and anti-inflammatory responses.

**Conclusion: **Our protocol allows large-scale production of EVs that are therapeutically effective in the pre–clinical model of PD.

## Graphical Abstract

**Graphical Abstract:**
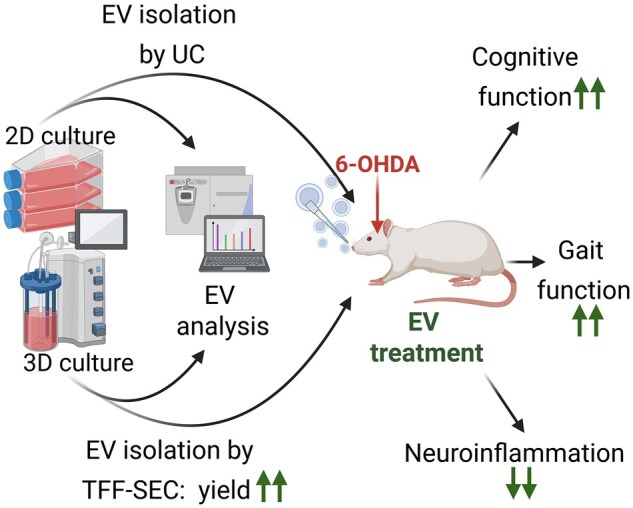

Significance statement This study establishes a scalable protocol for producing extracellular vesicles (EVs) from stem cells and demonstrates their therapeutic potential in a preclinical model of Parkinson’s disease. Intranasal delivery of therapeutic product generated by this approach markedly improved gait and cognitive function, supported dopaminergic neuron survival, and reduced neuroinflammation. By combining bioreactor-based culture with advanced isolation techniques, this strategy overcomes a major barrier to clinical translation by enabling large-scale production of EVs with consistent therapeutic activity.

## Introduction

Parkinson’s disease (PD) is the second leading progressive neurodegenerative disorder affecting millions of people worldwide.^1^ PD is characterized by the degeneration of dopaminergic neurons in the *substantia nigra* (SN) and the formation of Lewy bodies that represent cytoplasmic inclusions of insoluble alpha-synuclein aggregates.^2^ PD is primarily defined by motor dysfunction. However, cognitive impairments can manifest years before the onset of motor symptoms, and about 30% of patients develop dementia as the disease advances.^3^

At present, there is no effective cure for PD, treatments are symptomatic and do not halt the progression of neurodegeneration.^4^ Transplantation experiments in rodents^5^ and primates^6^ revealed that dopaminergic neurons derived from the induced pluripotent stem cells (iPSCs) survived, integrated into the host neural networks and improved specific symptoms of the disease. Clinical trials have already been initiated to test the safety and therapeutic efficacy of dopaminergic neurons derived from the allogeneic iPSCs in humans.^7^ However, stem cell therapy for PD patients will not be widely available in the foreseeable future.^8^ Therefore, new alternative therapies that effectively halt the progression of PD are in urgent demand.^8^

The potential use of extracellular vesicles (EVs) for therapeutic purposes attracted a great deal of interest in recent years.^9^^,^^10^ EVs represent a diverse group of lipid membrane-enclosed particles, that according to their origin and size, are classified into exosomes, microvesicles and apoptotic bodies.^11^ We and others have previously demonstrated that human stem cell-derived EVs exhibit neuroprotective properties *in vitro* and *in vivo.*^12–17^ Our group initially demonstrated that EVs derived from dental pulp cells of human exfoliated deciduous teeth (SHEDs) can suppress 6-hydroxydopamine (6-OHDA)-induced apoptosis in human dopaminergic neurons *in vitro.*^12^ In the following studies, we demonstrated the therapeutic efficacy of intranasal administration of EVs derived from SHEDs on a unilateral 6-OHDA medial forebrain bundle (MFB) rat model of PD.^13^ Intranasal therapy significantly improved gait and cognitive functions of PD-affected rats and normalized the expression of tyrosine hydroxylase (TH) in the SN and striatum.^13^ However, these therapeutic effects were transient, with cognitive improvements lasting up to 6 days and gait normalization persisting up to 10 days after discontinuation of treatment.^14^ These findings indicate that EVs can be used for the development of new minimally invasive treatment strategies against PD.

However, there are 2 major challenges that need to be solved before moving EV therapies toward clinical translation. First, therapeutic applications require large amounts of EVs that cannot be obtained by standard laboratory protocols. For example, to collect a necessary amount of EVs for our pre–clinical study with PD rats,^13^ we used supernatants from one hundred and six 150 cm^2^ T-flasks. Similar estimates were presented in other studies.^18^ Therefore, conventional laboratory-scale methods using standard cell cultivation on T-flasks and isolation of EVs by ultracentrifugation (UC) are not feasible for large-scale production of EVs. Optimized cell culture systems, such as 3D bioreactors, can significantly enhance cell and EV yield while reducing costs related to culture time, handling, and consumables.^18^ In addition, efficient EV isolation methods from large amounts of supernatants are needed. Tangential flow filtration (TFF) and chromatography techniques have been successfully employed for scaling up EV purification process.^19–22^ Cell culture supernatants processed by TFF are filtered and simultaneously concentrated using a peristaltic system equipped with high molecular weight cut-off membranes. The TFF method enables a substantial increase in the scale of EV production. For example, compared with UC, TFF has been reported to increase EV yield by approximately 7-fold^19^ or 5-fold.^23^ In addition, TFF has been shown to reduce protein impurities relative to UC.^23^ However, other studies using cryo-electron microscopy have demonstrated that EV fractions obtained by TFF may still be contaminated with protein aggregates and lipid droplets.^21^ For this reason, additional purification steps, such as size-exclusion chromatography (SEC) have been proposed to further improve EV purity.^20^^,^^22^^,^^24^

Second, it is well-known that parent cell type, its physiological state and culture conditions determine the molecular composition and therapeutic properties of EVs.^25^ Cell culturing parameters such as confluency, passage number, medium composition, and 2D or 3D culture can dramatically affect the biochemical composition, release and functional properties of the EVs.^26^ Many studies demonstrated that 3D culture profoundly affects proteomic,^27^ transcriptomic^28^ and metabolomic^29^ profiles of the EVs. Therefore, it is critically important that every step during the development of a new scale-up protocol should be followed by the molecular fingerprinting and validation of therapeutic efficacy using adequate *in vivo* models.

In the present study, we developed a new protocol for scaling up EV production and isolation from SHEDs for the treatment of PD. Our newly developed protocol increased the scale of EV production by 463-fold. More importantly, EVs produced by the new protocol significantly improved gait and cognitive functions, normalized TH expression and suppressed neuroinflammation in PD rats. Our results underscore the potential of large-scale EV production and emphasize the therapeutic advantages of EV-based interventions in neurodegenerative diseases.

## Methods

Additional methods can be found in the Supplemental materials and methods section.

### Bioreactor culture

**Figure 1.**
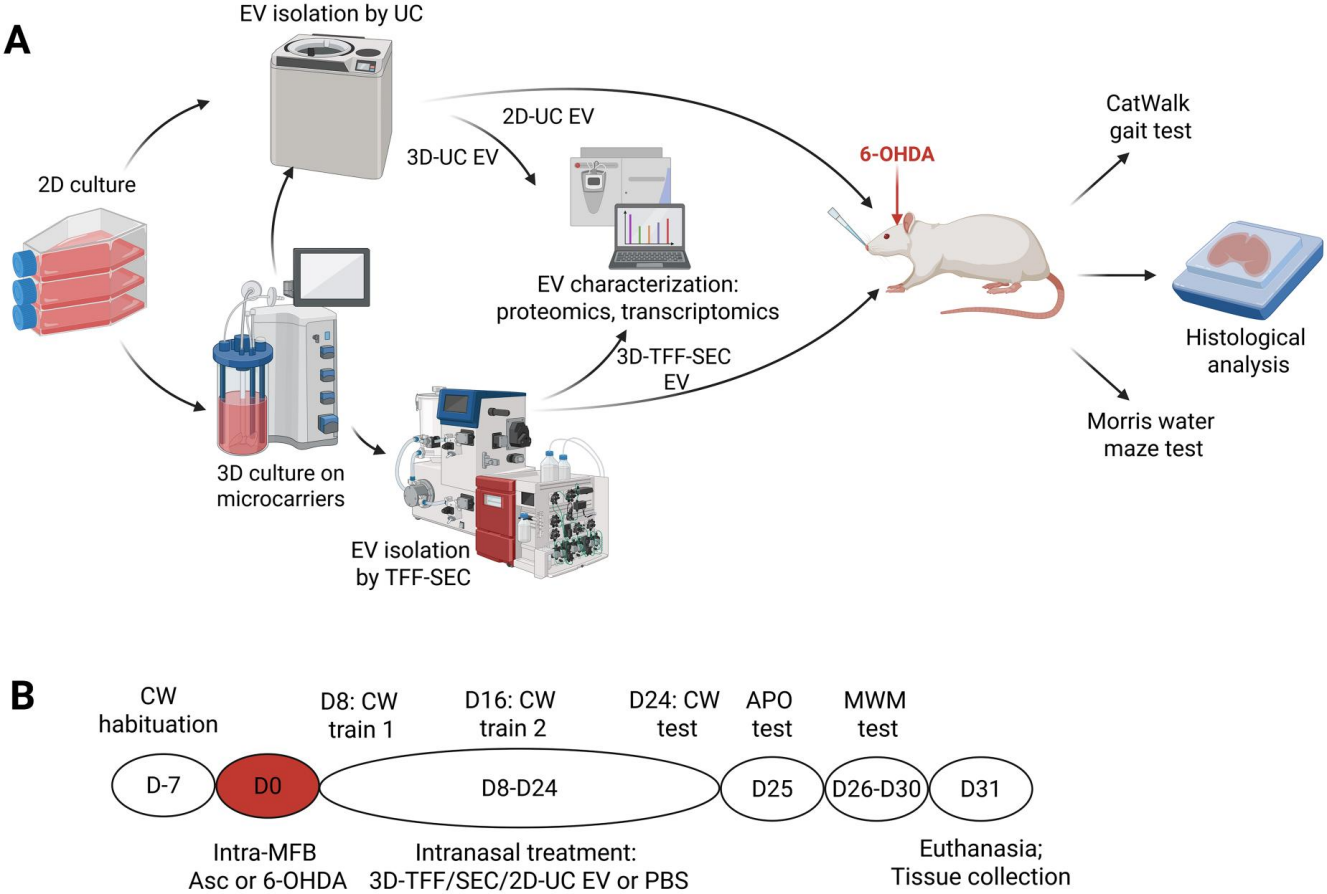
Workflow of EV production, characterization, and experimental design for *in vivo* testing in the 6-OHDA rat model of Parkinson’s disease. (A) SHEDs were cultured in either 2D T-flasks or expanded in 2D and transferred to a 3D bioreactor system. EVs were isolated from 2D cultures using differential ultracentrifugation (2D-UC EVs), and from 3D cultures using either ultracentrifugation (3D-UC EVs) or a combination of tangential flow filtration and size exclusion chromatography (3D-TFF-SEC EVs). All EV types were subjected to proteomic and transcriptomic profiling. 2D-UC and 3D-TFF-SEC EVs were selected for *in vivo* testing in a unilateral 6-OHDA rat model of Parkinson’s disease. EVs were administered intranasally, and functional outcomes were assessed using CatWalk gait analysis and the Morris water maze test. Following behavioral testing, rats were euthanized and brains were collected for histological analysis. Created in BioRender. https://BioRender.com/x66nce0. (B) Experimental design of the animal study. 6-OHDA, 6-hydroxydopamine; APO, apomorphine; Asc, ascorbate; CW, CatWalk; D, day; EVs, extracellular vesicles; MWM, Morris water maze.

We used a human immortalized SHED cell line (Lenti-hTERT-2A-CDK4). To obtain sufficient cell numbers for microcarrier inoculation, SHEDs were first expanded in 150 cm^2^ T-flasks (Figure 1).

### Microcarrier inoculation

Cytodex-1 microcarriers (Cytiva, 17044802) were suspended in Cellartis MSC Xeno-Free basal medium containing Cellartis MSC Xeno-Free supplement (Takara Bio, Y50200), 100 U/mL penicillin, and 100 μg/mL streptomycin. The suspension was kept at 37 °C for approximately 1 hour before inoculation. The cells were suspended in the serum-free medium and seeded onto microcarriers at a density of 10 000 cells/cm^2^ in a 2L Applikon bioreactor. The bioreactor parameters were controlled to maintain a temperature of 37 °C, 30% DO, 5% CO_2_, and a pH of 7.4. The microcarrier density was set to 10 cm^2^/mL, with a total medium volume of 600 mL. Stirring was maintained at 20 rpm overnight.

### Bioreactor expansion

Following inoculation, the stirring speed was increased to 100 rpm. Microcarriers were observed daily under a microscope to evaluate cell attachment and proliferation efficiency. EVs were harvested when approximately 80%-90% of the microcarrier surface area was covered with the cells (it took 4-5 days).

### Supernatant collection and cell reseeding

First, 90% of the culture supernatant was collected. The remaining cell-microcarrier suspension was transferred to test tubes to allow the microcarriers to settle. After removing the medium, the microcarriers were washed 3 times with PBS. Warm TrypLE Select (Gibco, 12563-029) was added, mixed, and incubated at 37 °C for 10 minutes. The resulting cell-microcarrier suspension was agitated using a serological pipette and filtered through 100 µm cell strainers (Th. Geyer, 7.696 769) placed in 50 mL test tubes. The strainers were washed 3 times with PBS. Separated cells were counted, resuspended in fresh medium, and reseeded onto newly prepared microcarriers under the same conditions.

### EV isolation using TFF combined with SEC

First, to remove detached cells and large debris, the conditioned culture medium was clarified by centrifugation at 700 × g for 10 minutes and 3000 × g for 10 minutes at 4 °C. The supernatant (500 mL) was collected and filtered through 0.22 µm vacuum filters (Corning, 430769).

The clarified medium was concentrated using a TFF system with 300 kDa cutoff hollow fiber filters (MidiKros 65 cm 300 kDa mPES, Repligen, D06-E300-05-N) in a Cogent µScale TFF system (Merck, Millipore). The process was conducted at a flow rate of 100.5 mL/minute (30% of maximum capacity) and a transmembrane pressure of 3.0 psi. A total of 500 mL of medium was concentrated to a final volume of 50 mL. This was followed by diafiltration with a 500 ml PBS buffer. The final concentrated and diafiltrated EV suspension volume was 50 ml.

The concentrated and dialyzed EV suspension was subjected to SEC using a HiScreen Capto Core 700 column (Cytiva, 17548115) on AKTA avant 25 preparative chromatography system (Cytiva). EVs were eluted with a PBS buffer, and 40 mL fractions were collected in 50 mL tubes. EV elution was monitored by UV absorption at 280 nm.

Purified EV suspensions were concentrated 10-fold using Amicon Ultra 100 kDa MWCO centrifugal filter units (Merck, Millipore, UFC9100) by centrifugation at 4000 × g at 4 °C. Final EV samples (5 mL) were aliquoted and stored at −80 °C for future use. Nanoparticle tracking analysis (NTA) of isolated EVs by both UC and TFF-SEC was performed with NanoSight LM10 (Malvern Panalytical, Malvern, UK).

### Animal experiments

#### Animals

Male Wistar Hannover rats (320 ± 20 g and 10-11 weeks old) were obtained from the Laboratory Animal Center, University of Tartu, Estonia. Experiments on animals were performed under the approval of the Animal Ethics Committee of the Food and Veterinary Service, Riga, Latvia (approval no. 144). All efforts were made to minimize animal suffering and reduce the number of animals used. The experiments were conducted in accordance with the EU Directive 2010/63/EU and local laws and policies on the protection of animals used for scientific purposes. Animals were housed in individually ventilated 2-level cages (GR1800, Tecniplast, Italy) in a controlled laboratory environment (temperature 24 °C ± 2°C, humidity 55%-65%, 12-hour light/dark cycle), 5 animals per cage with food and water provided ad libitum. Environmental enrichment in each cage included red polycarbonate cylinders, aspen bedding and aspen chewing blocks (Tapvei).

#### Experimental design

We used unilateral injection of 6-OHDA into the MFB of rats as a model of PD-type changes as described previously.^13^

**Figure 2.**
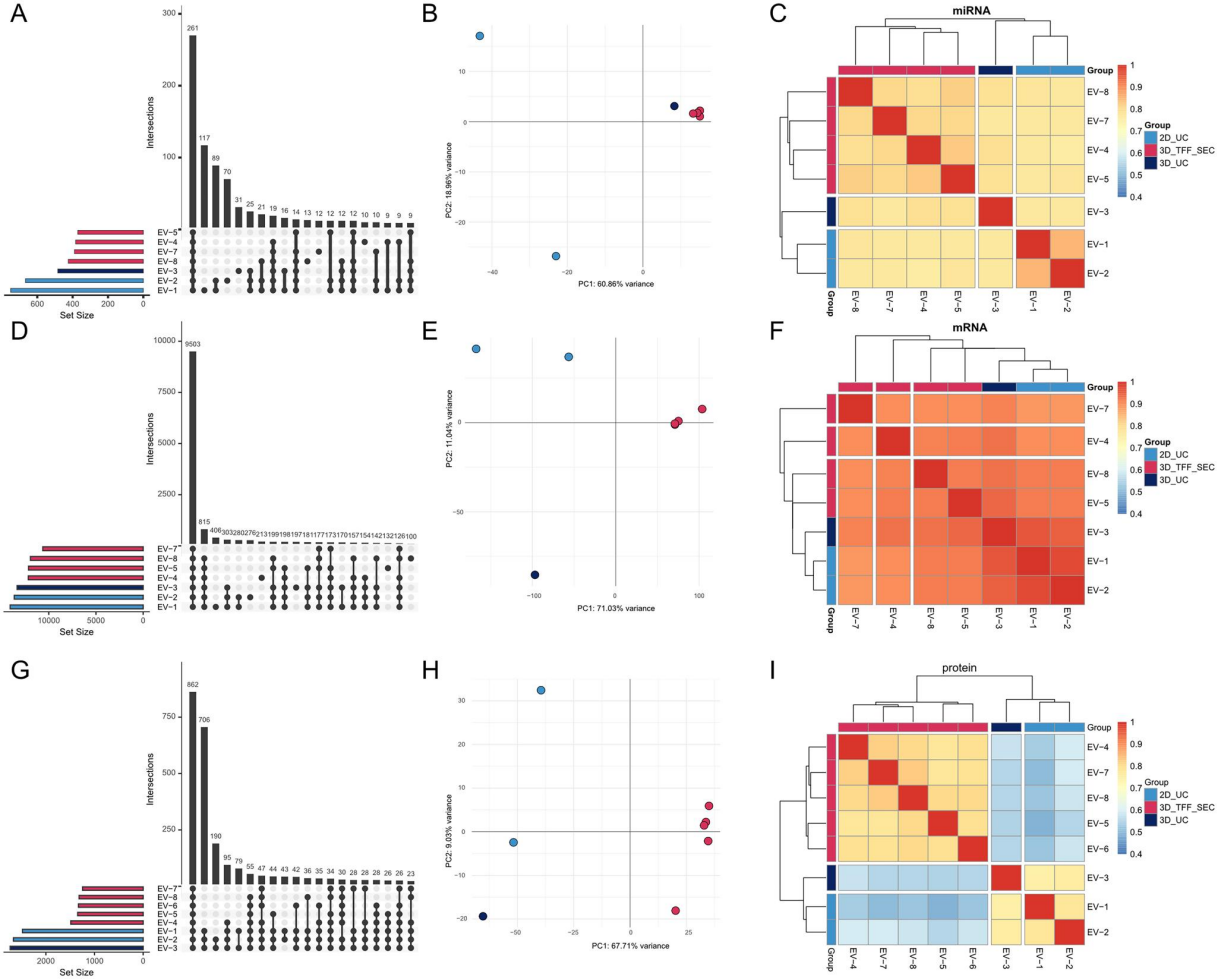
Transcriptomic and proteomic analysis to characterize the miRNA, mRNA, and protein content in extracellular vesicles (EVs) before (2D_UC), during (3D_UC), and after scale-up (3D_TFF-SEC). (A, D, G) Upset plots were used to compare the complexity in miRNA, mRNA, and protein profiles between the samples. (B, E, H) Principal component analysis (PCA) was performed to investigate the relationship between molecule patterns and cultivation and purification methods, respectively. PC1 loading exceeded 60% in all 3 analyses. (C, F, I) Spearman correlation heatmaps together with clustering of samples were performed to investigate the relationship between EVs cargo profiles based on the subset of consistently detected miRNAs, mRNA, and proteins.

Experimental design is shown in Figure 2. The rats were randomly divided into 6 groups (*n* = 9-12):

1. Group 1 (control) was injected with 0.1% Asc intra-MFB and PBS intranasally (i.n.);
2. Groups 2 and 3 were also injected with 0.1% Asc intra-MFB and then treated with either 3D-TFF/SEC (EVs produced and isolated using a new scaled-up protocol) or 2D-UC i.n.;
3. Group 4 (lesion) received 6-OHDA intra-MFB and PBS i.n.;
4. Groups 5 and 6 also received 6-OHDA intra-MFB, followed by either 3D-TFF/SEC or 2D-UC i.n.;

On D8, rats were administered EV suspension (Figure 1B) intranasally, in each nostril. A single dose of EVs contained 3.8 × 10^8^ EVs suspended in 10 µL of PBS. In total, during the treatment course, each rat received 17 daily EV treatments (D8-D24). Functional gait parameters (CatWalk, CW) and cognitive performance (Morris water maze, MWM) were assessed as described previously.^2^^,^^14^ On D31, animals were euthanized and immunohistochemical analysis was done.

#### Unilateral injection of 6-OHDA into the MFB

Rats underwent stereotaxic surgery on experimental day 0. Thirty minutes before the induction of general anesthesia (3%-3.5% isoflurane for induction and 2%-2.5% for maintenance), rats received imipramine (20 mg/kg) to protect adrenergic neurons against the 6-OHDA-induced lesions. Then, rats were placed on a stereotaxic frame (Stoelting Inc., USA), and freshly prepared 6-OHDA (20 μg in 3 μL of 0.1% Asc) or vehicle (Asc, 3 μL) were injected intra-MFB using the following coordinates: −4.4 mm anteroposterior, + 1.1 mm mediolateral, and −8.0 mm dorsoventral relative to the bregma, at a rate of 1 µL per min. The microinjection needle was left at the injection site for 3 minutes after the end of infusion to ensure distribution in the MFB. On post–lesion day 25, apomorphine was freshly dissolved in 0.9% NaCl and administered subcutaneously to rats at a dose of 0.2 mg/kg. Contralateral rotations were counted for 49 minutes to evaluate the hypersensitivity of the lesion induced by 6-OHDA.^30^ The calculation of contralateral rotations is depicted in Figure S2 (see online supplementary material for a color version of this figure).

#### Morris water maze test

Spatial learning and memory of healthy, PD-type model and EV-treated rats were assessed using the MWM test as described previously.^13^^,^^14^ Briefly, on experimental days 26-29, each animal underwent 4 trials (120 s maximum for each trial) per day. Animals were trained to find the hidden platform and stay on it for 15 seconds to stay on it to learn the location. On experimental day 30, animals underwent a probe trial, where the platform was removed from the pool, and each animal had to swim for 120 s.

#### CatWalk gait test

CW test was performed as described in Narbute et al.^13^^,^^14^ In short, all animals were trained 3 times: 7 days prior to 6-OHDA injection, on experimental day 8 (start of intranasal treatments) and experimental day 16 (halfway through the intranasal treatments). Habituation was done for 2 minutes before each training session. The final test was performed on experimental day 24. The CatWalk XT 11.0 quantitative gait analysis system (Noldus, The Netherlands) was used to assess the following parameters: stand, stride length, step cycle and duty cycle.^31^

### Statistical analysis

Behavioral and immunohistochemical results were averaged for each experimental group and presented as mean ± standard deviation (S.D.) values. Apomorphine-induced rotations were analyzed by Student’s *t*-test. For CW data, repeated-measure analysis of variance (RM-ANOVA) was done to compare gait outcomes between groups. In MWM, RM-ANOVA was performed to determine changes within and between groups during training, whereas 1-way ANOVA was utilized for MWM probe trial and quantitative immunohistochemical data. For all behavioral and immunohistochemical assessments, Holm-Sidak’s post‐hoc test was applied. Graph Pad Prism software version 9.0 (Graph Pad Software, Inc.) was used for data analysis. In all cases, *P*-values ≤ 0.05 were deemed significant.

## Results

### Comparison of EV yields obtained using standard and scale-up protocols

NTA revealed that in both samples, the main population of EVs peaks at around 100-130 nm. 2D-UC EVs exhibited a broader size distribution, including a greater proportion of larger particles with small peaks at 239, 335, and 459 nm, while 3D-TFF/SEC EVs were more uniform in size. Western blot confirmed that both types of EVs expressed key EV markers such as HSP70, syntenin-1 and CD63 (Figure S1A and B, see online supplementary material for a color version of this figure).

The use of a 3D bioreactor system for SHED culture on microcarriers, combined with subsequent EV isolation by UC (3D-UC), demonstrated a remarkable 20.3-fold increase in EV yield compared to the standard 2D culture approach using UC (2D-UC). Moreover, further purification of EVs using TFF followed by SEC (3D-TFF-SEC) resulted in an additional 22.8-fold increase in EV yield. When combined, the optimized 3D culture system and advanced purification protocol achieved an impressive 463-fold increase in overall EV production compared to the conventional 2D culture and UC method, as shown in Figure S1D (see online supplementary material for a color version of this figure). EV yields, as determined by NTA, were normalized to the growth surface area to ensure accurate comparisons across culture conditions.

### Comparison of protein, mRNA, and microRNA cargo between different EV preparations

To assess the impact of purification (UC vs. TFF-SEC) and cultivation (2D vs. 3D), a systematic analysis of the EV-associated miRNA, mRNA, and protein cargo was performed. The resulting data sets for each molecule type were investigated for their (1) complexity (number of identified proteins, mRNAs, and miRNAs), (2) patterns in molecule abundance using principal component analysis (PCA), and (3) reproducibility using Spearman correlation analysis (Figure 2).

The microRNA data (Figure 2A-C) showed that overall, >700 microRNAs were detected, of which 270 were found in all samples (Figure 2A). The complexity was higher in 2D_UC EV samples compared to TFF, with 276 miRNAs only detected in 2D_UC but not 3D_UC or 3D_TFF_SEC. PCA analysis showed that miRNA expression patterns in EVs harvested from 3D cultivated cells were similar, independent of the purification method, while 2D_UC EV exhibited marked differences in miRNA profiles (Figure 2B), which was confirmed by Pearson correlation analysis using the common set of 270 miRNAs (Figure 2C). This demonstrates that miRNA changes are subject to change when cultivation parameters are changed.

**Figure 3.**
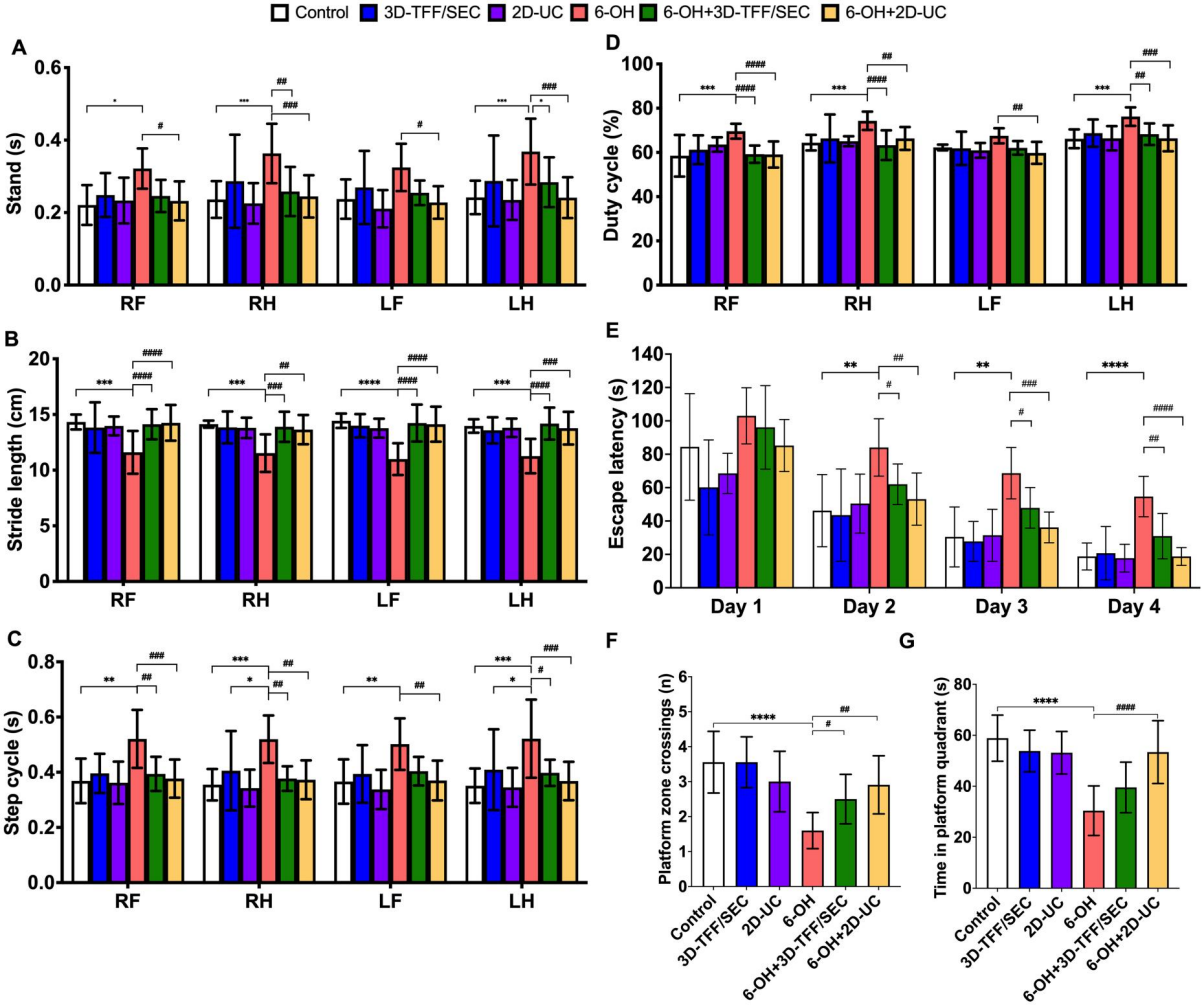
Intranasal treatment with extracellular vesicles (EVs) improves gait performance, spatial learning, and memory of 6-hydroxydopamine (6-OHDA)-injected rats. Parameters included standing time (A), stride length (B), step cycle duration (C), and the percentage of duty cycle (D). Spatial learning was assessed during 4 training days (E), whereas spatial memory—during the probe trial on day 5 as the amount of target zone crossings (F) and time spent in the target quadrant (G). Data shown as mean values ± SD (*n* = 9-12 animals per group). RF, right front; RH, right hind; LF, left front; LH, left hind paw. *P ≤ .05, **P ≤ .01, ***P ≤ .001, and ****P ≤ .0001 versus Control; ^#^*P* ≤ .05, ^##^*P* ≤ .01, ^###^*P* ≤ .001, and ^####^*P* ≤ .0001 versus 6-OH.

The mRNA data in EVs (Figure 3D-F) showed high uniformity across all samples. mRNA complexity was high, with 13902 detected mRNAs of which 9503 were detected in all samples. 2D_UC and 3D_UC samples exhibited slightly higher complexity, and PCA analysis indicates that mRNA patterns differ between UC and TFF-SEC purified EVs, however, Pearson Correlation analysis showed highly reproducible mRNA patterns across all EV samples. This demonstrates that mRNA profiles in EV preparations are only subject to small changes during the scale-up of EV production.

The protein data in EVs (Figure 3G-I) showed a different pattern. Overall, 2457 proteins were detected, of which 862 were present in all 8 EV preparations. Interestingly, protein complexity was highest in 2D_UC and 3D_UC samples, with 1018 proteins only detected in these groups but not in 3D_TFF_SEC. Vice versa, no proteins could be identified that are exclusively present in 3D_TFF_SEC but not in 3D_UC or 2D_UC. PCA analysis showed that protein patterns are highly reproducible in 3D_TFF_SEC samples, but different from 2D_UC and 3D_UC samples. Pearson correlation between the set of 862 overlapping proteins confirmed this result. This indicates that the change in EV purification from UC to TFF-SEC has a strong impact on the protein content in EV preparations.

In conclusion, our results demonstrate that culture conditions and isolation method differentially affect proteomic and miRNA cargo of EVs: when compared with UC, isolation using TFF/SEC substantially reduced the complexity of proteomic cargo of EVs, whereas culture conditions (2D versus 3D) affected miRNA, but not mRNA and proteomic content of the EVs.

### Comparison of the therapeutic efficacy of EVs in 6-OHDA rats

#### Intranasal administration of EVs improves gait parameters of 6-OHDA rats

We observed marked impairments in all studied gait parameters of 6-OH group rats in the CW test. First, 6-OH group rats stood significantly longer between steps on their right front (*P* ≤ 0.05), right hind (*P* ≤ 0.001), and left hind (*P* ≤ 0.001) paws compared to Control group animals (Figure 3A). In 6-OH group rats treated with 3D-TFF/SEC, the stand time was significantly shorter (*P* ≤ 0.01 for the right hind and *P* ≤ 0.05 for the left hind paw). In the 6-OH + 2D-UC group, the stand time of all paws was shorter compared to that of the 6-OH group rats (*P* ≤ 0.05 for both front paws, *P* ≤ 0.001 for both hind paws). Second, 6-OH group animals also had significantly shorter stride lengths in all paws (*P* ≤ 0.001 for the right front and both hind paws, *P* ≤ 0.0001 for the left hind paw; Figure 3B) compared to controls. Those 6-OH group rats that received intranasal 3D-TFF/SEC showed markedly longer stride length in all paws (*P* ≤ 0.001) when compared to the 6-OH group. 6-OH + 2D-UC group animals also exhibited a significant increase in stride length in all paws (*P* ≤ 0.0001 for both front paws, *P* ≤ 0.01 for both hind paws). Third, the step cycle of 6-OH group rats was markedly prolonged in all paws (*P* ≤ 0.01 for both front paws, *P* ≤ 0.05 for both hind paws) compared to the Control group (Figure 3C). This parameter was significantly shorter in the 6-OH + 3D-TFF/SEC group, namely in both right paws (*P* ≤ 0.001) and the left hind paw (*P* ≤ 0.05). Moreover, 6-OH + 2D-UC group animals had significantly shorter step cycles in all paws (*P* ≤ 0.01 vs. 6-OH). Finally, the duty cycle percentage of all paws except the left front paw in the 6-OH group was substantially increased (*P* ≤ 0.001, Figure 3D). Rats from the 6-OH + 3D-TFF/SEC group showed significantly lower duty cycle when compared to 6-OH group animals (*P* ≤ 0.0001 for both right paws, *P* ≤ 0.01 for the left hind paw). A similar decrease in duty cycle was observed in 6-OH + 2D-UC group rats (*P* ≤ 0.01 vs. 6-OH).

#### Intranasal administration of EVs ameliorates deficits in spatial learning and memory of 6-OHDA rats

Spatial learning of 6-OH group rats in the MWM test was significantly impaired, as they had longer escape latency on days 2 (*P* ≤ 0.01), 3 (*P* ≤ 0.01) and 4 (*P* ≤ 0.0001) compared to Control (Figure 3E), while 6-OH + 3D-TFF/SEC group rats demonstrated significantly shorter latency to 6-OHDA group (*P* ≤ 0.05 on day 2 and 3, *P* ≤ 0.01 on day 4). Rats from the 6-OH + 2D-UC group also showed markedly shorter escape latency on these days when compared to the 6-OHDA group (*P* ≤ 0.01 on day 2, *P* ≤ 0.001 on day 3 and *P* ≤ 0.0001 on day 4).

In the probe trial on day 5, rats from the 6-OH group also had significantly fewer platform zone crossings (*P* ≤ 0.0001, Figure 3F) and time spent in the platform quadrant (*P* ≤ 0.0001, Figure 3G) compared to controls. Rats from 6-OH + 3D-TFF/SEC and 6-OH + 2D-UC groups crossed the platform zone more times than the 6-OHDA group animals (*P* ≤ 0.05 in 6-OH + 3D-TFF/SEC and *P* ≤ 0.01 in 6-OH + 2D-UC group).

#### Intranasal administration of EVs preserves nigrostriatal density of TH in 6‐OHDA rats

**Figure 4.**
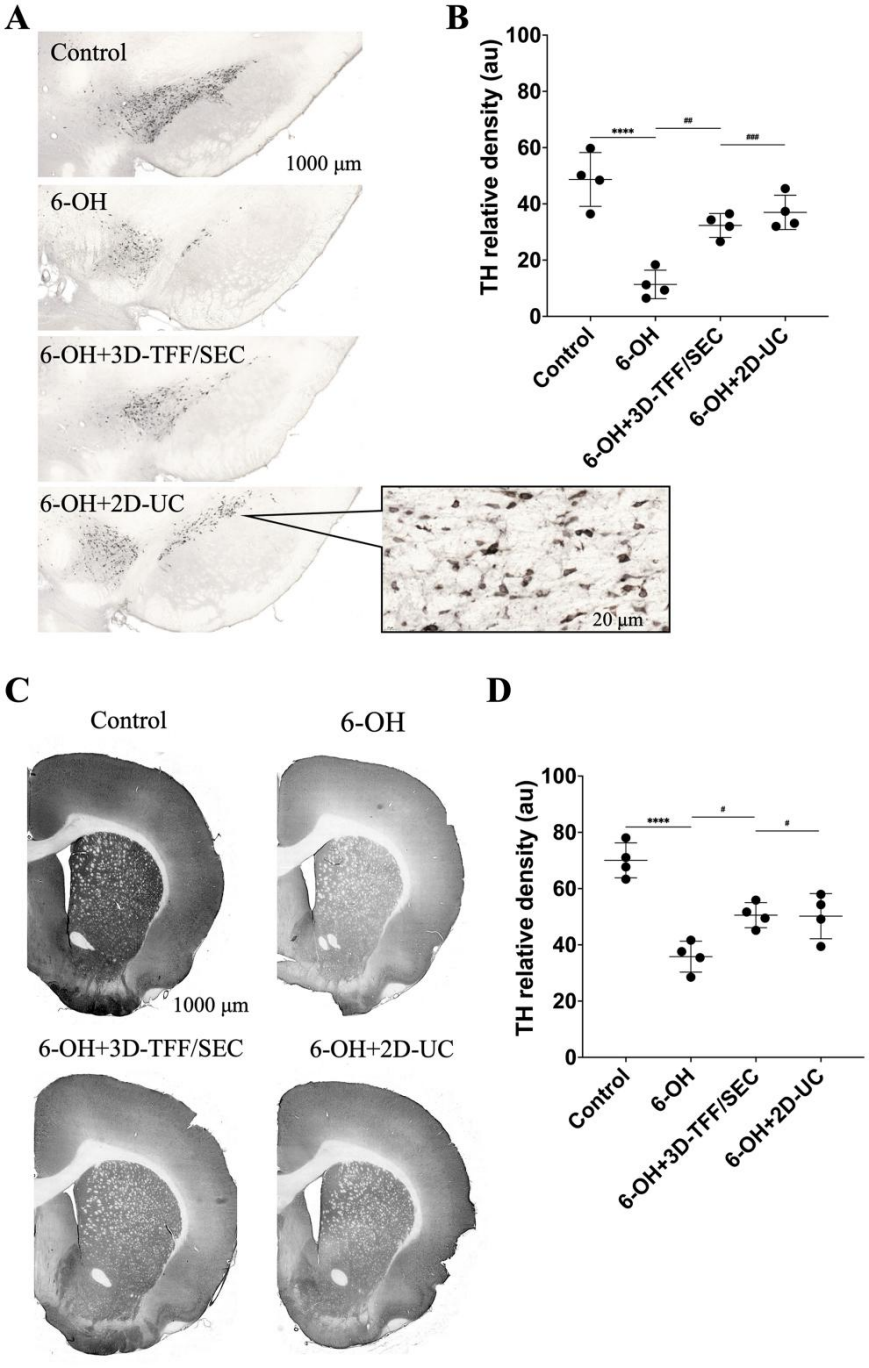
Intranasally administered EVs preserve the nigrostriatal optical density of tyrosine hydroxylase (TH) in 6-hydroxydopamine (6-OHDA)-injected rats. TH density in the striatum is shown in (A) and substantia nigra in (C). Densitometry bars show optical density of TH in the striatum (B) and substantia nigra (D). Data shown as mean values ± SD (*n* = 4 per group). ^#^*P* ≤ .05, ^##^*P* ≤ .01, ^###^*P* ≤ .001 and ****P ≤ .0001 versus 6-OH.

A substantial decrease in TH density was observed in the SN (*P* ≤ 0.0001, Figure 4A and B) and striatum (*P* ≤ 0.001, Figure 4C and D) of rats injected with 6‐OHDA compared to controls. Compared to 6-OHDA-injected rats, rats that received EVs exhibited increased TH density in the SN (*P* ≤ 0.01 in 6-OH + 3D-TFF/SEC and *P* ≤ 0.001 in 6-OH + 2D-UC group) and in the striatum (*P* ≤ 0.05 in both 6-OH + 3D-TFF/SEC and 6-OH + 2D-UC group).

#### Intranasal administration of EVs decreases astro- and microgliosis in 6‐OHDA rats

**Figure 5.**
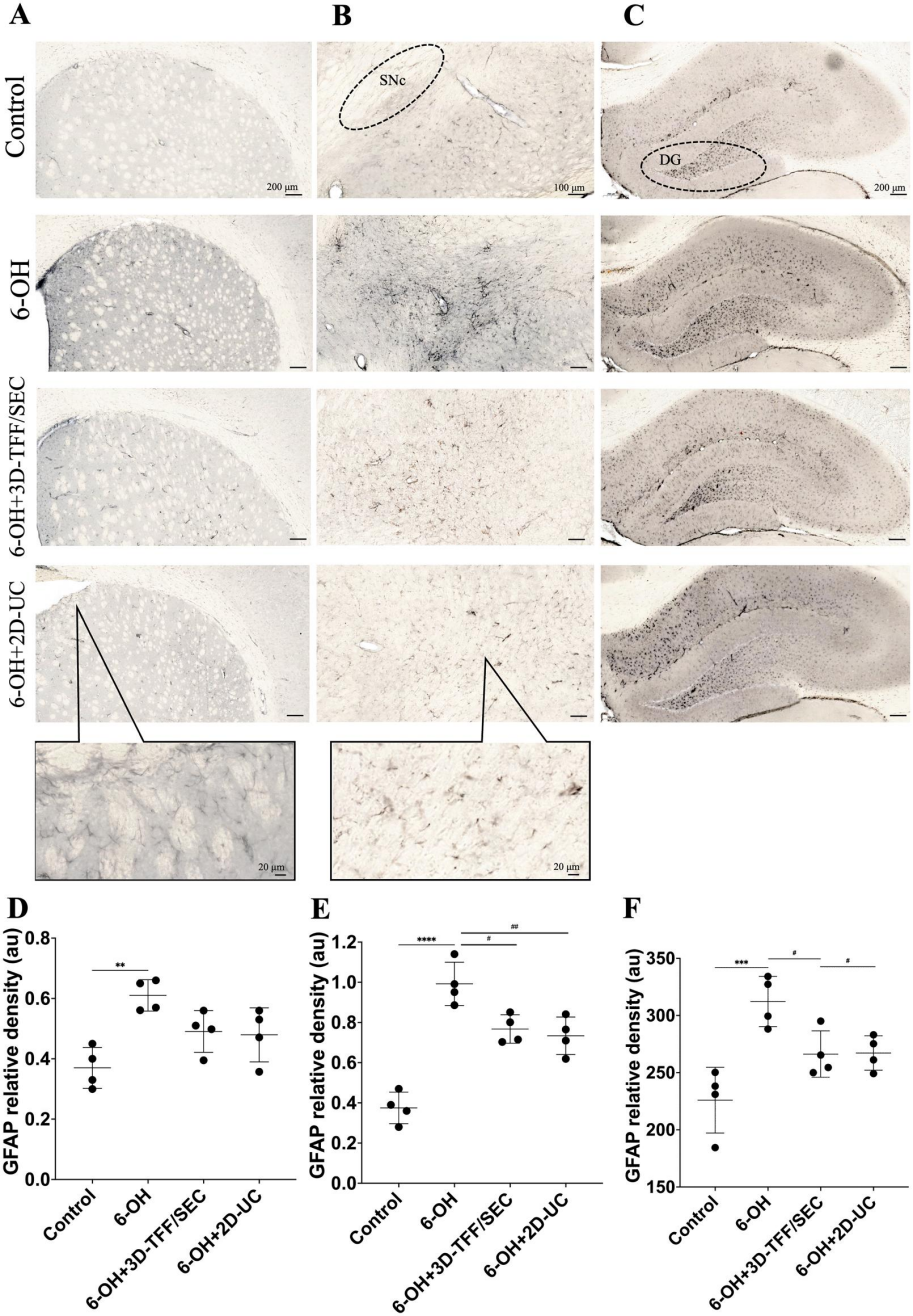
Extracellular vesicles (EVs) administered intranasally decrease the density of GFAP in the *substantia nigra* (SN) and hippocampal dentate gyrus (DG), but not the striatum of 6-hydroxydopamine (6-OHDA)-injected rats. Representative images show GFAP staining in the striatum (A), SN (B), and DG (C), whereas histograms (D, E, F) respectively show the quantification of GFAP density in the striatum, SN and DG. Data shown as mean values ± SD (*n* = 4 per group). **P ≤ .01, ***P ≤ .001, and ****P ≤ .0001 versus Control; ^#^*P* ≤ .05 and ^##^*P* ≤ .01 versus 6-OH.

The density of GFAP was increased in the striatum (*P* ≤ 0.01, Figure 5A and D) and markedly in the SN (*P* ≤ 0.0001, Figure 5B and E) of 6-OH group rats compared to controls. 6-OHDA-injected animals that were treated with intranasally administered EVs showed a decrease in GFAP density in the SN, but not striatum, compared to 6-OH group rats (*P* ≤ 0.05 in 6-OH + 3D-TFF/SEC and *P* ≤ 0.01 in 6-OH + 2D-UC group).

The density of astroglial protein GFAP was markedly elevated in the hippocampal dentate gyrus (DG) of 6-OHDA group rats (*P* ≤ 0.001, Figure 5C and F) in comparison to controls. Those 6-OHDA-injected animals that were treated with i.n. EVs showed a decrease in GFAP density compared to 6-OH group rats (*P* ≤ 0.05 in both 6-OH + 3D-TFF/SEC and in 6-OH + 2D-UC group).

**Figure 6.**
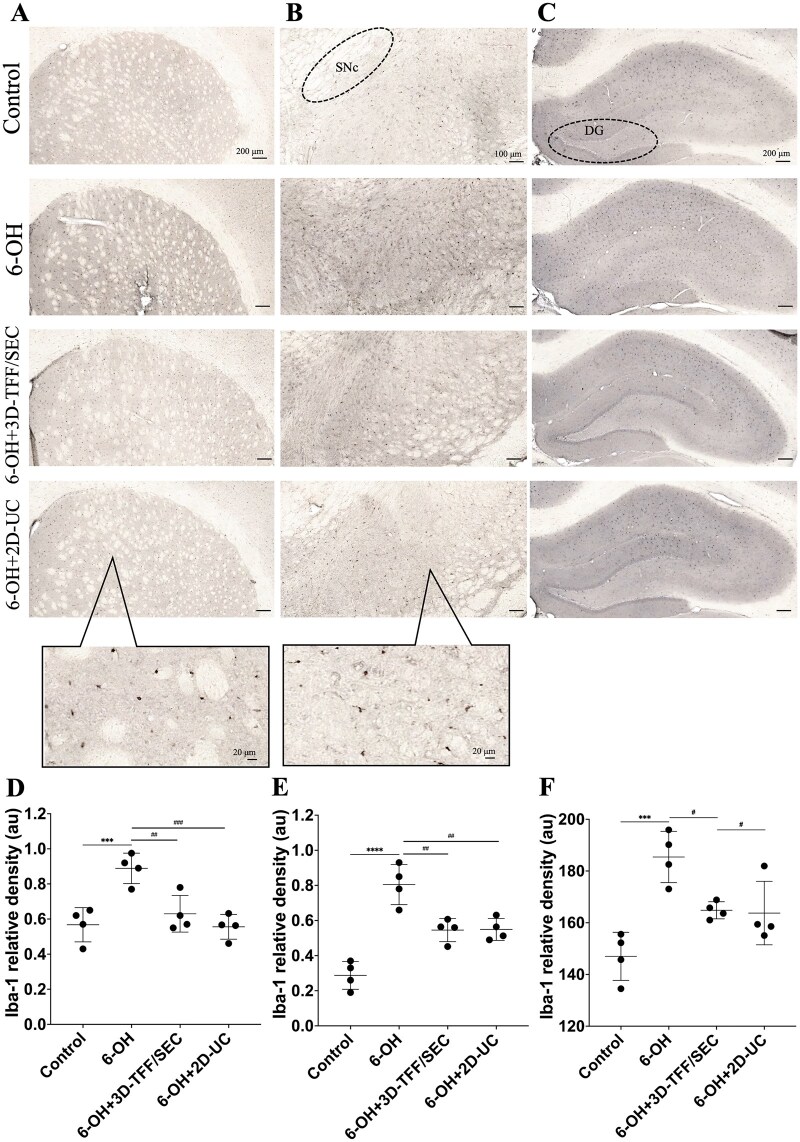
Intranasally administered extracellular vesicles (EVs) lower Iba-1 density in the striatum, *substantia nigra* (SN) and hippocampal dentate gyrus (DG)of 6-hydroxydopamine (6-OHDA)-injected rats. Representative images show Iba-1 staining in the striatum (A), SN (B), and DG (C), whereas histograms (D, E, F) respectively show the quantification of Iba-1 density in the striatum, SN and DG. Data shown as mean values ± SD (*n* = 4 per group). ***P ≤ .001 and ****P ≤ .0001 versus Control; ^#^*P* ≤ .05, ^##^*P* ≤ .01, and ^###^*P* ≤ .001 versus 6-OH.

The density of microgliosis protein Iba-1 was increased significantly in the striatum (*P* ≤ 0.001, Figure 6A and D) and SN (*P* ≤ 0.0001, Figure 6B and E) of lesion group rats in comparison to controls. In both treated groups, 6-OH + 3D-TFF/SEC and 6-OH + 2D-UC, the relative density of Iba-1 was significantly lower in the striatum (*P* ≤ 0.01 in 6-OH + 3D-TFF/SEC and *P* ≤ 0.001 in 6-OH + 2D-UC groups) and SN (*P* ≤ 0.01 in both 6-OH + 3D-TFF/SEC and 6-OH + 2D-UC groups) compared to the values in 6-OH group rats.

Iba-1 density was also increased significantly in the hippocampal DG of 6-OH group rats (*P* ≤ 0.0001 vs. Control, Figure 6C and F). It was significantly reduced, however, in the 6-OH group rats that received i.n. 3D-TFF/SEC and 2D-UC (*P* ≤ 0.05 for both 6-OH + 3D-TFF/SEC vs. 6-OH and 6-OH + 3D-TFF/SEC vs. 6-OH).

#### Intranasal administration of EVs decreases IL-1β expression in the SN of 6‐OHDA rats

**Figure 7.**
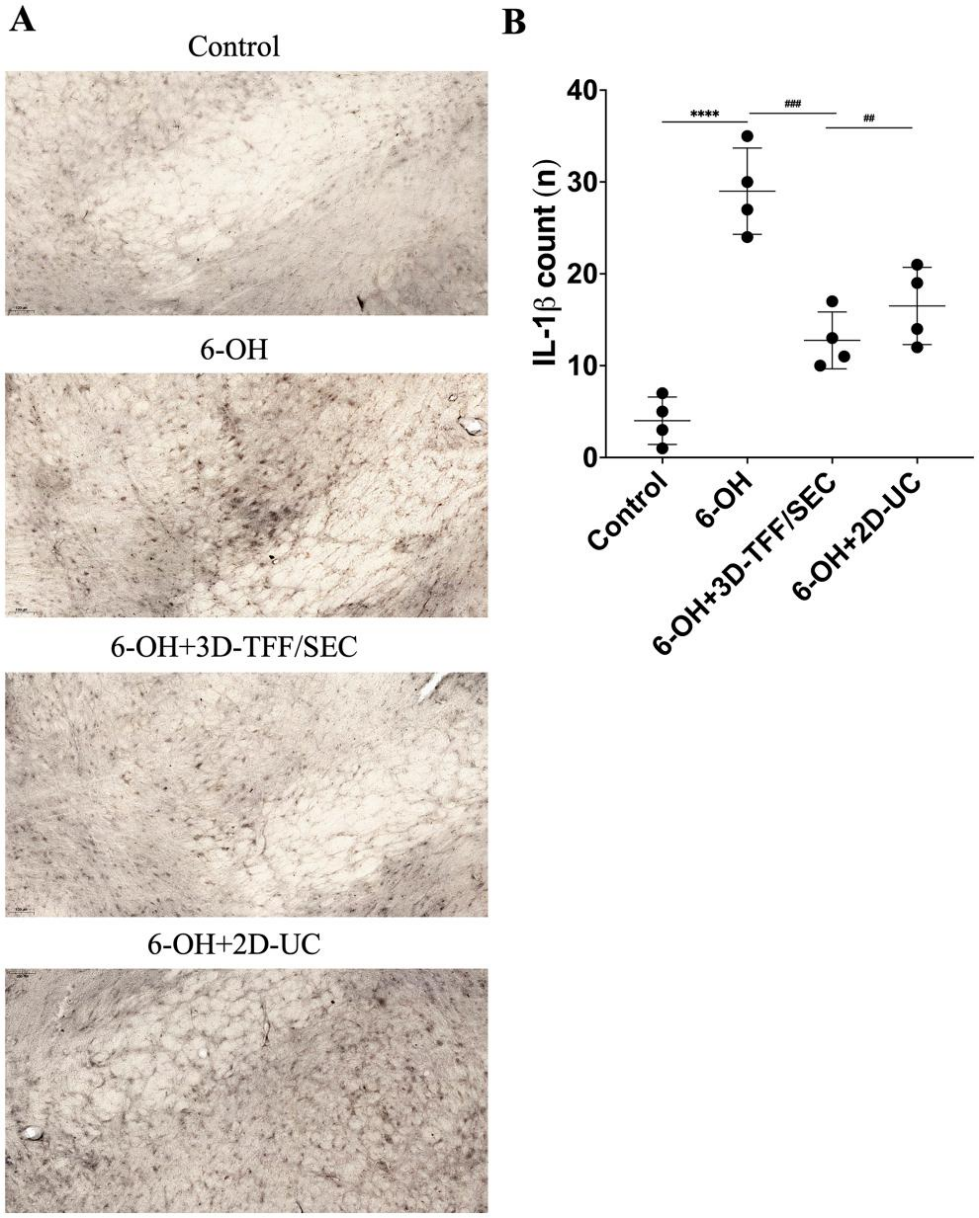
6-Hydroxydopamine (6-OHDA) rats treated with intranasal extracellular vesicles (EVs) demonstrate decreased IL-1β count in the SN. Microphotographs (A) depict IL-1β-positive cells in the SN at ×200 magnification, whereas the histogram (B) shows the analysis between groups. Data shown as mean values ± SD (*n* = 4 per group). ^##^*P* ≤ .01, ^###^*P* ≤ .001 and ^****^P ≤ 0.0001 versus 6-OH.

Cells positive for inflammasome-related marker IL-1β were counted in the SN of controls, 6-OHDA-injected and 6-OH group animals that received EVs. Marked elevation of IL-1β-positive cells was observed in the SN of 6-OH group rats in comparison to controls (*P* ≤ 0.0001). Rats treated with EVs demonstrated a substantial decrease in the IL-1β expression in the SN (*P* ≤ 0.001 in 6-OH + 3D-TFF/SEC and *P* ≤ 0.01 in 6-OH + 2D-UC groups) when compared to 6-OH group animals (Figure 7).

## Discussion

Scaling up EV production is a prerequisite for clinical translation. However, cell culturing parameters and isolation methods can significantly alter the molecular composition and, consequently, the therapeutic efficacy of EV preparations. In the present study, we developed a new scale-up protocol based on microcarrier culture of immortalized human SHEDs in a spinning bioreactor and subsequent isolation of EVs from the supernatants using a 2-step TFF/SEC protocol. We then performed extensive proteomic and RNA profiling to assess the impact of culture conditions and method of isolation on the molecular cargo of EVs.

Interestingly, proteomic and transcriptomic analyses revealed that EVs from both 2D-UC and 3D-UC groups displayed remarkably similar protein and mRNA profiles, showing that microcarrier culture did not significantly affect the protein and mRNA cargo. Comparison of proteomic profiles between 3D-UC and 3D-TFF/SEC groups revealed that EV isolation using TFF/SEC substantially reduced the complexity of the EV proteome (Figure 2G). This is in agreement with previous studies showing the superiority of the SEC method over UC in the removal of protein contaminants from EV preparations [20, 27-29]. Unexpectedly, most proteins not presented in the 3D-TFF/SEC group can be associated with GO terms such as Endosome membrane (GO: 0010008), Vesicle (GO: 0031982), Extracellular exosome (GO: 0070062), Extracellular vesicle (GO: 1903561) and Vesicle membrane (GO: 0012506) (Figure S3, see online supplementary material for a color version of this figure) These findings suggest that part of the vesicular fraction of EV secretome was lost during TFF/SEC isolation. This result can be at least in part explained by the fact that we did not perform 0.22 µm filtration of supernatants collected from the 3D microcarrier cultures prior to differential UC. It is also possible that high centrifugal forces during UC can damage and disrupt some EVs, leading to precipitation of large EV protein aggregates and membrane fragments enriched in the EV proteins.

In contrast to proteomic data, microRNA analysis demonstrated that culture conditions, but not isolation method, affected the microRNA cargo of EVs (Figure 3A-C). When compared to 3D UC, 2D-UC EV preparations contained >270 additional microRNAs. Thus, 3D microcarrier cultures increased EV yields but reduced the complexity of the microRNA repertoire. By contrast, neither culture conditions nor isolation method significantly affected mRNA fingerprints in different EV preparations.

Having demonstrated that both culture conditions and isolation methods have a major impact on the molecular cargo of the EVs, we next compared the therapeutic efficacy of both EV products in an animal model of PD. We have previously demonstrated that intranasal administration of EVs derived using standard lab scale protocol (2D-UC) significantly improved gait and cognitive functions in the unilateral 6-OHDA MFB rat model of PD.^13^^,^^14^ In the present study, we used the same experimental model for direct comparison of therapeutic efficacy between EVs derived using standard and newly developed scale-up protocol (3D-TFF/SEC). Our results demonstrate that both EV preparations markedly improved gait and cognitive functions in the PD rats (Figure 3). Interestingly, EVs derived from 2D-UC cultures more effectively improved cognitive parameters than those obtained using 3D-TFF-SEC. Immunohistochemical analyses further supported our findings, demonstrating that both EV preparations preserved TH density in the striatum and SN decreased astro- and microgliosis and pro-inflammatory cytokine expression in PD-model rats (Figures 4-7). EVs from 2D-UC cultures produced a slightly greater reduction in Iba-1—positive microglia and better preservation of nigrostriatal TH density compared with EVs from 3D-TFF/SEC cultures, although both treatments significantly improved outcomes relative to the lesion group. The differences between 2D-UC and 3D-TFF/SEC EVs, while measurable, were modest in magnitude, indicating that both culture systems produce EV populations containing therapeutically active molecular cargo. Although 2D-UC derived EVs showed slightly stronger neuroprotective effects, 3D culture systems are more practical for large-scale clinical use due to their higher EV yields and greater compatibility with scalable good manufacturing practice (GMP)—compliant production.

Ideally, developers of EV therapeutic product should provide regulatory authorities with the following information: (1) what biological substance present in the EV preparation is responsible for the observed therapeutic effects of the EVs; (2) the amount of therapeutically active biological substance present in the particular preparation of the EVs; (3) what is the mechanism of action (MoA) of the therapeutic molecule and how it depends on the EV dose.^32^^,^^33^ However, these requirements may be challenging due to the enormous molecular complexity of the EVs. In this study, we identified 1700 proteins that were present in both EV preparations. Assuming a high probability that therapeutically active molecules should be most abundantly expressed, the list of proteins, miRNAs and mRNAs can be narrowed. Using this approach, we created a list of the 170 most abundantly expressed proteins (Table S1) and identified several candidates that could be potentially responsible for the *in vivo* neuroprotective action.

6-OHDA is a neurotoxin which is selectively accumulated in the dopaminergic neurons and, after autooxidation, induces excessive production of reactive oxygen species (ROS), oxidation of proteins, DNA and lipids, respiratory redox failure and mitochondrial dysfunction.^34^ We therefore propose that EVs suppress the neurotoxic action of the 6-OHDA by reducing oxidative stress in dopaminergic neurons of the SN. Indeed, proteomic analysis identified several proteins that were abundant in both EV preparations and were previously described as potent suppressors of oxidative stress in PD. Among them was Apolipoprotein D (ApoD), a lipocalin transporter of small hydrophobic molecules, such as arachidonic acid, steroid hormones and sphingolipids.^35^ The expression of ApoD was increased in glial cells surrounding dopaminergic neurons in the brains of PD patients.^36^ Furthermore, several studies demonstrated that ApoD secreted by EVs from astrocytes promotes survival of dopaminergic neurons by preventing the rise of brain lipid peroxides under oxidative stress.^35–38^ EVs also expressed high levels of glutathione S-transferase Pi 1 (GSTP1) and glutathione S-transferase omega 1 (GSTO-1) proteins. Both enzymes catalyze the conjugation of glutathione (GSH) to a wide variety of electrophilic and toxic compounds, including products of lipid peroxidation and dopamine metabolism, thereby reducing oxidative stress.^39^ Importantly, both EV preparations also contained (although not in the top 10% of most abundant proteins) superoxide dismutase 1 (SOD1), catalase (CAT) and peroxiredoxin-2 (PRDX2). These enzymes are essential for neutralizing ROS and protecting dopaminergic neurons from oxidative damage in PD.^40^

Degeneration of dopaminergic neurons induces chronic activation of surrounding microglia, leading to inflammasome activation and increased IL1β production, which in turn accelerates the progression of neurodegeneration.^2^^,^^41^ Here, we show that EVs suppressed microgliosis and IL1β expression in the SN of 6-OHDA rats (Figures 6 and 7). We have recently demonstrated that EVs promote autophagy in human microglia.^42^ It has been shown that increased autophagy in microglia reduced the NLR family, pyrin domain containing 3 (NLRP3) protein expression, which in turn deactivated the NLRP3 inflammasome.^43^ We therefore propose that EV-induced suppression of inflammasome activation during 6-OHDA-induced injury can be at least partially mediated by increased autophagy in microglia. Proteomic analysis also identified high levels of anti-inflammatory proteins ANXA1, ANXA2 and ANXA5 in both EV preparations. ANXA1 helps maintain brain homeostasis by promoting the clearance of apoptotic cells and suppressing microglial activation.^44^ Furthermore, genetic variants in ANXA1 that impair its function are associated with increased risk of Parkinsonism.^45^ ANXA2 and ANXA5 both regulate autophagy and facilitate anti-inflammatory responses in the context of PD.^46^^,^^47^ Additionally, 5'-nucleotidase (CD73) was identified among the most abundant proteins. By converting pro-inflammatory ATP/AMP signals into anti-inflammatory adenosine, EV-associated CD73 functions as a powerful immunomodulatory.^48^ CD73 suppresses immune cell activation, reduces cytokine release, and limits tissue infiltration, protecting neural tissue against inflammatory damage.^49^

We also identified 51 miRNAs that were most abundantly expressed in both EV groups (Table S2). Several miRNAs from this list can be associated with anti-oxidative and anti-inflammatory responses in PD. miR-221-3p is known as a promoter of neuronal survival against oxidative stress by modulating the Akt signaling pathway in PD.^50^ miR-26b-5p has been linked to nicotine’s protective effects in PD.^51^ Exosomal miR-100-5p suppressed oxidative stress in dopaminergic neurons by targeting Nox4-ROS-Nrf2 axis in 1-methyl-4-phenyl-1,2,3,6-tetrahydropyridine (MPTP) mice model of PD.^52^ miR-223-3p is a negative regulator of NLRP3 inflammasome activity.^53^ Future studies are needed to obtain real gene expression data from the EV-treated and untreated 6-OHDA rat brain tissues to perform accurate miRNA target prediction. It is also worth mentioning that, according to some estimations, EVs exert their therapeutic actions through proteins rather than miRNAs.^54^ Nevertheless, specific sets of miRNAs presented in the therapeutically active EV preparations can be utilized as useful markers during clinical manufacturing.

Thus, cargo analysis of both 2D-UC and 3D-TFF/SEC EV preparations revealed that they carry many proteins and miRNAs that can be involved in the therapeutic action by suppressing oxidative stress and inflammatory response. However, the exact identification of molecule(s) responsible for the therapeutic effect is technically difficult and sometimes can be impossible. Also, given the enormous molecular complexity of the EVs, there is a high probability that, in most cases, the therapeutic properties of the EVs depend on the net effect of different cargo molecules rather than on the individual players. If this is the case, then searching for specific biological substance(s) responsible for the therapeutic action of EVs is impractical and can be counterproductive. In this respect, molecular fingerprinting of therapeutically active EV preparations used in this study can provide a useful alternative. This tactic could help to dispose of “molecular noise” and identify sets of proteins, RNA species and potentially lipids and metabolites that are enriched in the therapeutically active EV preparations. In the future, these repositories can be used during clinical manufacturing for the prediction of the therapeutic efficacy of different lots of EVs.

In this study, we demonstrated that intranasal treatment with EVs derived using our newly developed scale-up protocol (1) significantly improved gait and cognitive functions; (2) preserved dopaminergic neurons in the striatum and SN; and (3) decreased astrogliosis and suppressed neuroinflammation during 6-OHDA-induced neurodegeneration in rats. However, it should be noted that functional tests were performed a month after 6-OHDA injection, and IHC data only show one time point, that is, 1 week after the cessation of EV treatment (Figure 2). Thus, currently, we do not know the mechanism responsible for the neuroprotective actions of EVs during the 17-day-long treatment period. Future studies focusing on the gene expression patterns during the early, middle and late phases of EV treatment can provide useful information about the possible MoA of EVs.

## Conclusion

Despite the potential benefits, clinically useful EV-based therapies are still not available for patients. Currently, the lack of standardized and pre–clinically validated large-scale EV production protocols represents one of the bottlenecks slowing down the progression toward clinical translation. Our protocol allows large-scale production of EVs that are therapeutically effective in the pre–clinical model of PD. In the future, this protocol can be adopted for manufacturing under GMP conditions and clinical trials.

## Supplementary Material

szag024_Supplementary_Data

## Data Availability

Data supporting the findings of this study are available from the corresponding author upon reasonable request.

## Patent info

Patent application number GB 2416757.9.
