# Supplementary material for: Extracellular vesicles produced by a large-scale protocol are therapeutically effective in preclinical model of Parkinson’s disease
Source: Stem Cells Transl Med. 2026 Apr 29;15(5):szag024. doi: 10.1093/stcltm/szag024 (PMC13124282; doi:10.1093/stcltm/szag024)
Supplement: szag024_Supplementary_Data [file szag024_supplementary_data.zip › Supplemental_Material_R1-02-19 final.docx]

**SUPPLEMENTARY INFORMATION**

**Extracellular vesicles produced by a large-scale protocol are therapeutically effective in pre-clinical model of Parkinson’s disease**

**Large scale production of EVs for treatment of PD**

Agnė Pociūtė^1,2^, Virginijus Tunaitis^1,2^, Arūnas Žebrauskas^1,2^, Vladimirs Pilipenko^3^, Baiba Jansone^3^, Karina Narbute^4^, Marianne Pultar^5^, Matthias Hackl^5^, Augustas Pivoriūnas^1,2^

^1^Department of Stem Cell Biology, State Research Institute Centre for Innovative Medicine, LT-01102, Vilnius, Lithuania.

^2^Exosomica, LT-11127, Vilnius, Lithuania.

^3^Faculty of Medicine and Life Sciences, University of Latvia, Jelgavas 3, Riga, Latvia

^4^Latvian Biomedical Research and Study Center, Rātsupītes Iela 1, Riga, 1067, Latvia.

^5^TAmiRNA GmbH, 1110 Vienna, Austria

**Correspondence:** Augustas Pivoriūnas, PhD, ^1^ Department of Stem Cell Biology, State Research Institute Centre for Innovative Medicine, Santariškių 5, Vilnius, Lithuania, LT-01102. E-mail: [augustas.pivoriunas@imcentras.lt](mailto:augustas.pivoriunas@imcentras.lt).

**Supplemental materials and methods**

*Chemicals and Antibodies*

The following chemicals were purchased from Sigma-Aldrich (USA): apomorphine (A4393-1G), 3,3′-diaminobenzidine (DAB, D5905), DPX mountant (06522), bovine serum albumin (BSA, A2058) and anti-TH antibody (T2928), Triton X-100 (X100), 6-OHDA (H116) and xylene (214736). Antibodies against GFAP (ab68428) were from Abcam (UK), IL1β (PA5-105048), ANXA1 (55018-1-AP), goat anti-mouse IgGs (31430) and goat anti-rabbit IgGs (31460) – from Thermo Fisher Scientific (Germany), Iba-1 (019-741) was from Wako (Japan), HSP70 (610608) – from BD Biosciencies (USA), MFG-E8 (sc-271574), MHC-I (sc-55582), CD63 (sc-5275), TSG101 (sc-7964) and Syntenin-1 (sc-100336) from Santa Cruz Biotechnology (USA). Apomorphine was dissolved in saline prior to the injection. 6-OHDA was freshly dissolved in 0.1% ascorbic acid (Asc) before intracerebral injections.

*Isolation, culture and immortalization of SHEDs*

The SHED cells were isolated from the exfoliated deciduous tooth of a child according to a previously published protocol [12, 13]. Cells were immortalized by overexpression of hTERT and CDK4 genes using lentiviral vector transduction (Lenti-hTERT-2A-CDK4-Puro (Applied Biological Materials)) according to the manufacturer’s instructions. Briefly, SHED cells (4 passages) were plated in a 6-well plate at a density of 1.8 × 10^5^ cells per well. The next day, cells were treated with 2 ml transduction mixture, containing 1 ml culture medium, 1 ml lentivirus solution (10⁶ IU/ml) and 8µg/ml Polybrene. After 8 hours, the transduction mixture was removed and replaced with a fresh one for overnight incubation. The next day, the viral media were changed to complete growth media. 48 hours later, the cells split into two 100 mm dishes. Selection of immortalized cells was performed using puromycin (1 µg/ml) for 4 days. The selected cells were analysed or stored in liquid nitrogen for future usage. The activity of overexpressed hTERT in the immortalized SHED cell line was analysed using the Quantitative Telomerase Detection Kit (Allied Biotech, Inc., Vallejo, CA) according to the manufacturer’s instructions.

Cells were cultured in DMEM-GlutaMAX medium containing 1 g/L D-glucose (Gibco, 21885025), supplemented with 10% foetal bovine serum (FBS, Gibco, 10500064), 100 U/ml penicillin, and 100 μg/mL streptomycin until they reached about 80% confluence. Cultures were maintained in a 37 °C incubator with a humidified atmosphere of 5% CO₂.

During subculturing, the culture medium was aspirated, and the cells were washed once with phosphate-buffered saline (PBS) solution. A 0.25 % trypsin-EDTA solution (Gibco, 25200-072) was then added, and the cells were incubated at 37 °C for 3-5 minutes to form a suspension. The trypsin-EDTA was neutralized with a double volume of culture medium, followed by centrifugation of the cell suspension at 500 × g for 5 minutes. The cells were distributed to new T-flasks for expansion and/or EV isolation or used to inoculate microcarriers.

For EV isolation from 2D cultures, cells were seeded at 4000-5000 cells/cm^2^ in T150 flasks and cultivated in Cellartis MSC Xeno-Free basal medium containing Cellartis MSC Xeno-Free supplement (Takara Bio, Y50200), 100 U/ml penicillin, and 100 μg/ml streptomycin until 80-90 % confluent (3-4 days).

*EV isolation using differential ultracentrifugation (UC)*

EVs were isolated using a modified differential centrifugation protocol based on a previously described protocol ^27^. All centrifugation steps were carried out at 4 °C. Supernatants collected from cells cultured in serum-free medium were centrifuged sequentially at increasing speeds: 300 × g for 10 minutes, 2,000 × g for 10 minutes, and 20,000 × g for 30 minutes. The resulting supernatants were then subjected to ultracentrifugation at 100,000 × g for 70 minutes using a Sorvall LYNX 6000 ultracentrifuge with a T29-8×50 rotor in Oak Ridge centrifuge tubes (Thermo Fisher Scientific, Rochester, NY). Pellets were washed in 40 ml of sterile, ice-cold PBS and ultracentrifuged again at 100,000 × g for 70 minutes under the same conditions. Final EV pellets were resuspended in sterile PBS and stored at -80 °C.

*EV isolation using tangential flow filtration combined with size-exclusion chromatography (TFF/SEC)*

*Tangential flow filtration*

Clarified conditioned medium (CM) was concentrated using a tangential flow filtration (TFF) system equipped with 300 kDa cut-off hollow fiber filters (MidiKros, 65 cm, 300 kDa mPES; Repligen, D06-E300-05-N) mounted on a Cogent µScale TFF system (Merck Millipore). Filtration was performed at a flow rate of 100.5 mL/min (30% of maximum capacity) with a transmembrane pressure not exceeding 3.0 psi. Prior to sample processing, the TFF column was rinsed with 3.7 L of Milli-Q water and sanitized by recirculation with 1 L of 0.5 M NaOH for 1 h. The column was then equilibrated with 185 mL of 0.01 M PBS buffer before loading the clarified conditioned medium. A total volume of 500 mL was concentrated to 50 mL. Diafiltration was performed using 10 volumes (500 mL) of 0.01 M PBS to remove residual contaminants and exchange the buffer. The sample was further concentrated to 25 mL and combined with 10 mL of residual volume recovered from the column and tubing, along with 15 mL of PBS used to flush the system, yielding a final EV suspension volume of 50 mL in PBS. Following sample processing, the TFF column was rinsed with 800 mL of Milli-Q water, filled with 800 mL of 0.5 M NaOH, and recirculated until 200 mL remained, followed by an additional 30 min of recirculation. The column was then washed with 200 mL of Milli-Q water, filled with 200 mL of 20% ethanol, recirculated for 5 min, disconnected, and stored at 4 °C.

***Size-exclusion chromatography***

The concentrated and diafiltrated EV suspension was further purified by size-exclusion chromatography (SEC) using a HiScreen Capto Core 700 column (Cytiva, 17548115) on an ÄKTA avant 25 preparative chromatography system (Cytiva). Prior to chromatography, system channels were flushed with Milli-Q water, sanitized by incubation with 0.5 M NaOH for 60 min, and washed again with Milli-Q water. The SEC column was washed with three column volumes (CV) of Milli-Q water, sanitized with three CV of 0.5 M NaOH for 30 min, washed again with three CV of Milli-Q water, and equilibrated with 0.01 M PBS buffer until the pH reached 7.4. The EV sample was loaded onto the column at a flow rate of 2 mL/min, with the pressure limit set to 0.5 MPa. EV-containing flow-through fractions were monitored by UV absorbance at 280 nm and collected into 50 mL tubes. For column regeneration, internalized small molecular impurities (<700 kDa) were eluted using 2 M NaCl until a stable UV baseline was achieved, followed by reverse-flow cleaning with 1 M NaOH in 30% isopropanol. The column was then washed with three CV of Milli-Q water and filled with 20% ethanol for long-term storage.

Purified EV suspensions were concentrated tenfold using Amicon Ultra centrifugal filter units (100 kDa MWCO; Merck Millipore, UFC9100) by centrifugation at 4,000 × g at 4 °C. Final EV preparations (5 mL) were aliquoted and stored at −80 °C until further use.

*Protein isolation and Western Blot analysis*

Cells were detached from T-flasks or microcarriers, and the resulting suspension was centrifuged at 1,000 × g for 5 minutes. The cell pellet was washed with PBS, followed by another centrifugation at 1,000 × g for 5 minutes. Cells were lysed in Pierce RIPA buffer (Thermo Fisher Scientific) supplemented with protease inhibitor cocktail (MP biomedicals) by incubating the samples on ice with agitation for 30 min. To remove cell debris, the lysates were centrifuged at 18,000 × g for 20 minutes at 4 °C. EVs were lysed by mixing the EV suspension with Pierce RIPA buffer (supplemented with protease inhibitor cocktail) at a 1:1 ratio and incubating the mixture on ice with agitation for 20 minutes. Protein concentrations were measured using the Pierce BCA Protein Assay Kit (Thermo Fisher Scientific, 23225) according to the manufacturer’s protocol.

For Western blot analysis, lysates were diluted in 6× Laemmli sample buffer and denatured at 95 °C for 5 minutes. Equal amounts of protein from each lysate were subjected to sodium dodecyl sulphate polyacrylamide gel electrophoresis (SDS-PAGE) in a 4–10% gel, run on a Mini-PROTEAN Tetra cell apparatus (Bio-Rad). Separated proteins were transferred onto a PVDF membrane using the semi-dry Trans-Blot Turbo transfer system (Bio-Rad).

The PVDF membrane was blocked in 5% bovine serum albumin (BSA; Applichem) in PBS-Tween 20 (0.18%, PBS-T) for 1 hour at room temperature (RT) on a platform rocker. It was then incubated overnight at 4 °C with primary antibodies diluted in 5% BSA/PBS-T. After primary antibody incubation, the membrane was washed three times with PBS-T and then incubated with a horseradish peroxidase-conjugated secondary antibody (1:2000 dilution in PBS-T; Thermo Scientific) for 1 hour at RT on a platform rocker. Following another round of washing, immunoreactive bands were detected using the Clarity ECL Western blotting substrate (Bio-Rad) and visualized on a ChemiDoc MP imaging system (Bio-Rad).

*Transmission electron microscopy (TEM)*

TEM of EVs was performed according to the previously reported protocol {Thery, 2006 #79} with some modifications. Briefly, EVs in PBS were fixed in 2% paraformaldehyde (PFA) for 40 minutes on ice. Formvar-carbon-coated copper grids were floated on a 10-µl drops of fixed EV suspension for 20 minutes at RT. Then, the grids were washed with PBS and floated on a 30-µl drops of 1% glutaraldehyde for 5 minutes at RT, then again washed eight times by transferring from one drop of distilled water to another. Samples were contrasted on drops of Uranyless (em-grade, 11000) for 5 minutes at RT in the dark. Finally, grids were air-dried for 5 minutes. The samples were then analysed with the transmission electron microscope FEI Tecnai G2 F20 X-TWIN.

***EV and cellular proteomic analysis***

*Lysis of cell samples*

The MS analysis was performed at the DTU Proteomics Core. 50 μL of Lysis buffer (6M Guanidinium Hydrochloride, 10 mM TCEP, 40 mM CAA, 50 mM HEPES pH 8.5) was added to each sample along with a 3 mm tungsten carbide bead (Qiagen). Samples were treated twice in the TissueLyser II homogenizer (Qiagen), going from 3-30 Hz in 60 seconds. Beads were removed, and samples were transferred to clean Eppendorf LoBind tubes. Samples were boiled at 95 °C for 5 minutes, followed by sonication on high for 5 x 60 seconds on and 30 seconds off in a Bioruptor Pico sonication water bath (Diagenode) at 4°C. Samples were centrifuged at 10,000 RCF for 10 minutes, and supernatants were transferred to clean Eppendorf Protein LoBind tubes.

*Lysis of EV samples*

High volume samples concentrated in an Eppendorf Concentrator plus to 30μL. 50 μL of Lysis buffer was added to each sample, after which they were boiled at 95°C for 5 minutes, followed by sonication on high for 5 × 60 seconds on and 30 seconds off in a Bioruptor Pico sonication water bath (Diagenode) at 4°C.

*Digestion of all samples*

Protein concentrations were determined by BCA Rapid Gold (Thermo Scientific), and 20 μg of protein was taken forward for digestion. The sample volume was normalized between samples with additional lysis buffer and diluted 3x with digestion buffer (10 % Acetonitrile in 50 mM HEPES pH 8.5), and 400 ng of LysC (MS-grade Wako) was added. Samples were incubated for 3.5 hours at 37°C, shaking at 750 RPM. After LysC digestion, samples were diluted to a final 10x in digestion buffer and digested with 200 ng Trypsin (MS-Grade, Sigma Aldrich) overnight at 37°C, shaking at 750 RPM. Digestion was stopped by adding 2 % trifluoroacetic acid (TFA) to a final concentration of 1 %. The resulting peptides were desalted on a SOLAμ SPE plate (HRP, Thermo). Between each application, the solvent was spun through by centrifugation at 350 RCF. For each sample, the filters were activated with 200 μL of 100 % Methanol, then 200 μL of 80 % Acetonitrile, 0.1 % formic acid. The filters were equilibrated twice with 200 μL of 1 % TFA, 3% Acetonitrile, after which the sample was loaded. After washing the tips twice with 200 μL of 0.1 % formic acid, the peptides were eluted into clean Eppendorf Protein LoBind tubes using 40 % Acetonitrile, 0.1% formic acid. The eluted peptides were concentrated in an Eppendorf Speedvac and reconstituted in 12μL A* buffer (2 % Acetonitrile, 1 % TFA) and nano dropped on a DeNovix DS-11 FX+ to determine peptide concentration*.*

*MS analysis*

Peptides were loaded onto a 2cm C18 trap column (ThermoFisher 164946), connected in-line to a 15cm C18 reverse-phase analytical column (Thermo EasySpray ES904) using 100% Buffer A (0.1 % Formic acid in water) at 750 bar, using the Thermo EasyLC 1200 HPLC system, and the column oven operating at 30 °C. Peptides were eluted over a 70 minute gradient ranging from 10 % to 60 % of Buffer B (80 % acetonitrile, 0.1% formic acid) at 250 nl/minute, and the Orbitrap Exploris instrument (Thermo Fisher Scientific) was run in DIA mode with FAIMS ProTM Interface (ThermoFisher Scientific) with CV of -45 V. Full MS spectra were collected at a resolution of 120,000, with an AGC target of 300 % or maximum injection time set to ‘auto’ and a scan range of 400–1000 m/z. The MS2 spectra were obtained in DIA mode in the orbitrap operating at a resolution of 60.000, with an AGC target 1000 % or maximum injection time set to ‘auto’, a normalised HCD collision energy of 32. The isolation window was set to 6 m/z with a 1 m/z overlap and window placement on. Each DIA experiment covered a range of 200 m/z, resulting in three DIA experiments (400-600 m/z, 600-800 m/z and 800-1000 m/z). Between the DIA experiments, a full MS scan is performed. MS performance was verified for consistency by running complex cell lysate quality control standards, and chromatography was monitored to check for reproducibility.

*Data analysis*

Biogenity has contributed by performing the data analysis, including figures, tables, and reports. The raw files were analyzed using Spectronaut™ (version 17.4) spectra were matched against the human database from Uniprot. Dynamic modifications were set as Oxidation (M) and Acetyl on protein N-termini. Cysteine carbamidomethyl was set as a static modification. All results were filtered to a 1 % FDR, and protein quantitation was done on the MS1 level. Protein groups were inferred by IDPicker. The data was filtered so only proteins with 2 unique peptides and at least 60 % valid values across all of the samples, or at least 75 % within an experimental group were used in the downstream data analysis. Filtration was performed using the R programming language.

***EV and cellular miRNA and mRNA analysis***

*RNA extraction*

Total RNA was extracted from EV samples and cells using the miRNeasy Mini Kit (Qiagen, Hilden, Germany) with an on-column DNase I digest. For the EV samples, 7 µL glycogen (5 mg/mL) was added for enhanced precipitation. RNA was eluted in 30 µL nuclease-free water and stored at -80 °C until further analysis. For cell samples, the quality of extracted total RNA was assessed using the Agilent RNA 6000 Nano kit (5067-1511, Agilent Technologies, Waldbronn, Germany) for 2100 Bioanalyzer systems.

*Small RNA library preparation*

8.5 µL (EVs) or 100 ng (cells) total RNA were used as input for the generation of small RNA sequencing libraries. Libraries were generated with the RealSeq Biofluids library preparation kit (RealSeq Biosciences, CA, USA) according to the manufacturer’s instructions. To each sample, 1 µL miND® spike-in standards (TAmiRNA, Vienna, Austria) were added during the first step as described previously ^28^. Adapter-ligated libraries were amplified (20 cycles for EV samples; 18 cycles for cells) using barcoded Illumina reverse primers in combination with the Illumina forward primer. Library quality control was performed using DNA 1000 chips (Agilent Technologies, Waldbronn, Germany). All samples were pooled equimolarly and processed with the Blue Pippin system (Sage Science, MA, USA) using 3 % agarose size selection cassettes, following the manufacturer’s instructions (size range: 130-160 bp). Sequencing was performed on an Illumina NovaSeq SP Flowcell in SR100 mode.

*Whole-transcriptome sequencing library preparation*

6 µl total RNA from EVs and 10 ng total RNA from cells were used as input for the generation of whole-transcriptome sequencing libraries using the SMARTer stranded total RNA sequencing kit v3.0 pico (TakaraBio) according to the manufacturer's instructions. Pre-amplification was performed using 10 PCR cycles in steps 1 and 2, respectively. Library yield was assessed on the Bioanalyzer DNA high-sensitivity assay between 200 and 2000 bp. Libraries were pooled equimolarly and sequenced on a NovaSeq S4 in paired-end 150 mode (Illumina).

*Bioinformatics analysis of transcriptomics data*

Small RNA-sequencing data were analyzed using the miND® analysis pipeline ^29^ and evaluated with fastQC v0.11.9 ^30^ and multiQC v1.14 ^31^. Reads were adapter-trimmed, and quality filtered using cutadapt v3.3 (Martin, 2011). Mapping steps were performed with Bowtie v1.3.0 ^32^ and miRDeep2 v2.0.1.2 ^33^. Reads were initially mapped against the genomic reference GRCh38.p12 by Ensembl ^34^ allowing two mismatches and subsequently against miRBase v22.1 ^35^, filtered for microRNAs of hsa, allowing one mismatch. For a general RNA composition, non-microRNA mapped reads were mapped against RNAcentral v19.0 ^36^ and assigned to RNA species of interest.

Overall quality of the whole-transcriptomic next-generation sequencing data was evaluated automatically and manually using fastQC v0.11.8 and multiQC v1.7. Sequencing reads from all passing samples were adapter-trimmed and quality filtered with bbduk from the bbmap package v38.69 ^37^, removing sequencing adapters, low-quality bases, and short reads below a predefined length threshold. Reads were then aligned to the Homo sapiens reference genome GRCh38.p13 provided by Ensembl using STAR v2.7 ^38^. Gene expression quantification was carried out using Salmon v1.1 in alignment-based mode ^39^.

*Statistical Analysis*

Statistical analysis of NGS data (miRNA and whole-transcriptomic) and proteomics was conducted with R V4.0. Prior to unsupervised investigation and overlap analysis, whole-transcriptomic data were filtered for protein-coding genes only. Overlap analysis was conducted using the package UpSetR v1.4. Transcriptomics count data and log-transformed proteomics data were used for unsupervised exploration. Spearman correlation distances were calculated using rstatix v0.7.2. and clustered using the package pheatmap v1.0.12. Principal component analysis was performed using the pcaMethods v1.98.0 package.

*Immunohistochemical assays*

Immunohistochemical analysis was done according to a modified protocol described in ^13,14^. On experimental day 31, animals were deeply sedated using a ketamine and xylazine mixture (100 mg/kg and 10 mg/kg, respectively) and transcardially perfused. Whole brains of animals were post-fixed in 4 % PFA overnight and then cryoprotected in 30 % sucrose and 0.1 % sodium azide-containing solution for 48 hours.

For each brain, 30 μm thick coronal slices were obtained using a cryotome at -25°C (CM1850, Leica Biosystems, Nussloch, Germany): 6 sections each from the corpus striatum (AP plane -0.36 mm to -0.60 mm from bregma), SN (AP plane -4.80 mm to -5.04 mm from bregma) and hippocampus (AP -3.30 mm to -4.00 mm from bregma).

Sections were rinsed in 0.3% Tween®20-containing PBS (PBST), then incubated for 10 minutes at 95 °C in 0.01 M sodium citrate buffer (pH 6.0) and subsequently blocked in PBST containing 3 % BSA for 2 hours to decrease backstain formation. Afterwards, sections were incubated overnight at 4 °C in a 1 % BSA-PBST blocking solution containing primary antibodies against TH (1:1000), GFAP (1:500), Iba-1 (1:1000) or IL-1β (1:200). The next day, sections were rinsed and incubated in a blocking solution containing either goat secondary antibody against mouse IgGs (31430, 1:1000) or rabbit IgGs (31460, 1:1000) for 2 h at room temperature. Finally, sections were rinsed in PBST, stained in DAB solution for 5-10 minutes, washed in xylene and coverslipped using DPX mountant.

*Quantification*

The mounted brain sections were scanned using a Pannoramic Midi II Scanner (3DHISTECH, Budapest, Hungary) at ×200 magnification. The optical density of TH was determined in the striatum and substantia nigra (SN), whereas that of GFAP and Iba-1 staining – in the hippocampal dentate gyrus (DG). IL-1β-positive (IL-1β+) cells were counted in the SN. An observer blinded to the treatment of the animals performed all measurements in duplicate using the ImageJ software.

**Supplemental tables**

**Table S1.** Expression levels of top 10 % most abundant proteins across 2D-UC and 3D-TFF/SEC samples. The table contains expression values for proteins ranked in the top 10 % by mean abundance across all samples. Only features detected in at least one sample of both 2D-UC (n = 2) and 3D-TFF/SEC (n = 4) were included.

**Table S2.** Expression levels of top 10 % most abundant miRNAs across 2D-UC and 3D-TFF/SEC samples. The table contains expression values for miRNAs ranked in the top 10 % by mean abundance across all samples. Only features detected in at least one sample of both 2D-UC (n = 2) and 3D-TFF/SEC (n = 4) were included.

**Table S3.** Expression levels of top 10 % most abundant mRNAs across 2D-UC and 3D-TFF/SEC samples. The table contains expression values for mRNAs ranked in the top 10 % by mean abundance across all samples. Only features detected in at least one sample of both 2D-UC (n = 2) and 3D-TFF/SEC (n = 4) were included.

**Supplemental figures**


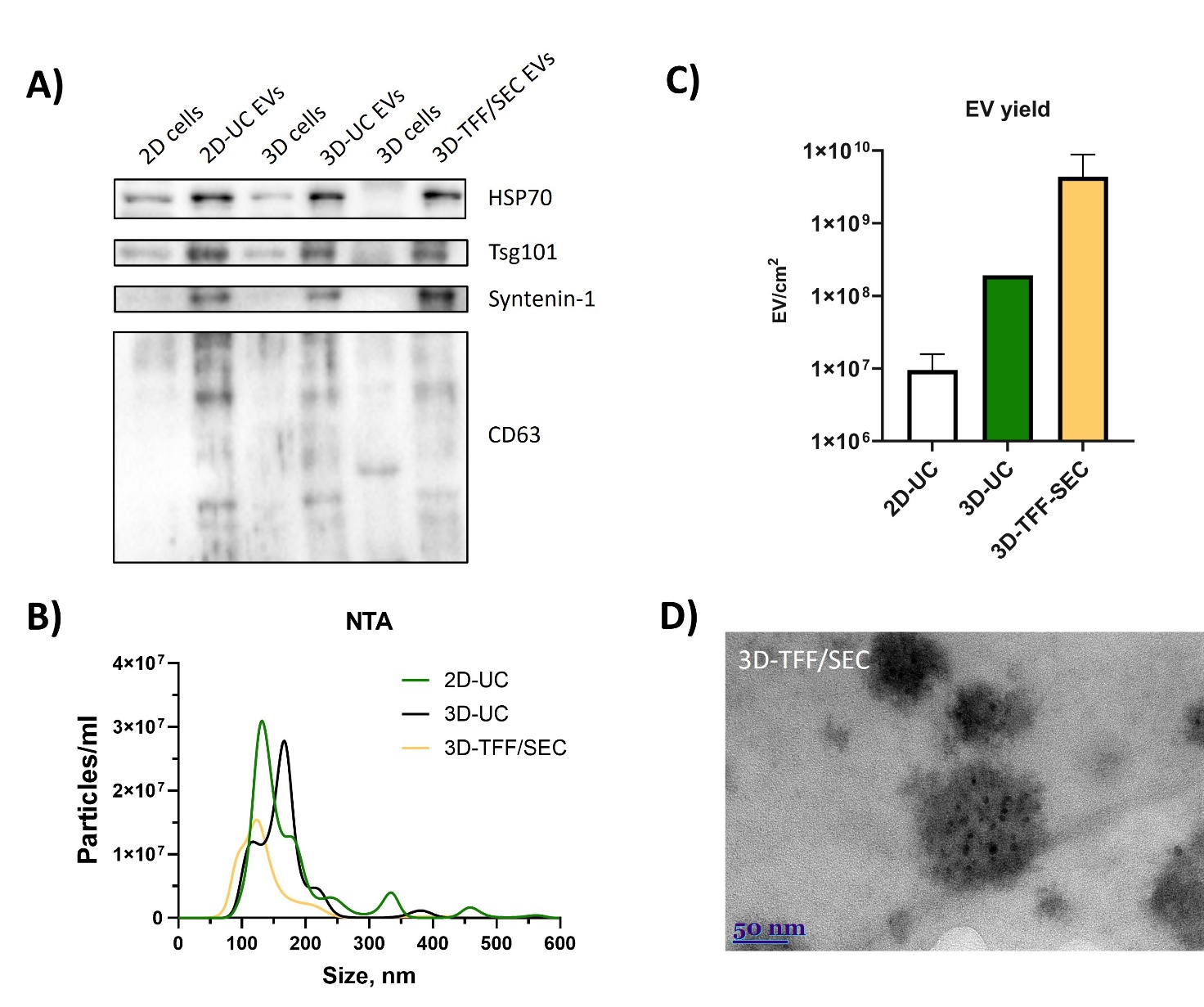


**Figure S1.** **EV quantification and characterization.** **A)** Cells and EV lysates were subjected to SDS-PAGE electrophoresis, blotted and the membrane was probed with antibodies against EV markers HSP70, TSG101, syntenin-1 and CD63. **B)** EV size and distribution. Nanoparticle tracking analysis (NTA) was performed using NanoSight LM10 instrument (Malvern Panalytical). For measurements samples were diluted: 2D-UC – 100 times, 3D-UC – 1500 times and 3D-TFF/SEC – 2500 times. **C)** Transmission electron microscopy of 3D-TFF/SEC EVs. **D)** Comparison of EV isolation methods by EV yield. The amount of EVs was normalized to growth surface area. Data is shown as mean + SD, n = 1-5.


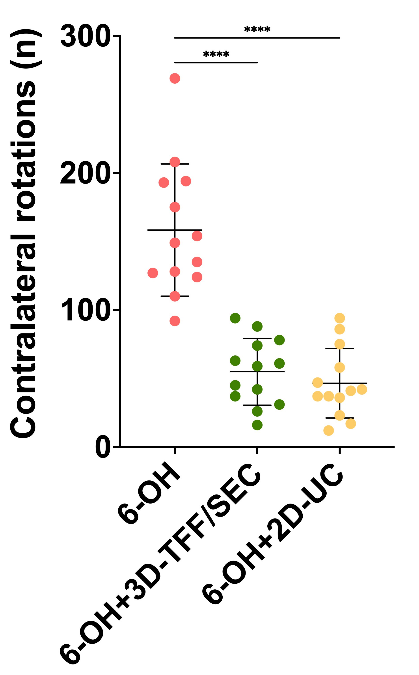


**Figure S2. Assessment of 6‐OHDA‐injected rats' performance in the APO test** **(n=13/group).** Number of contralateral rotations was counted on post-lesion day 25.  *****p* ≤ 0.0001 vs. 6-OH group.


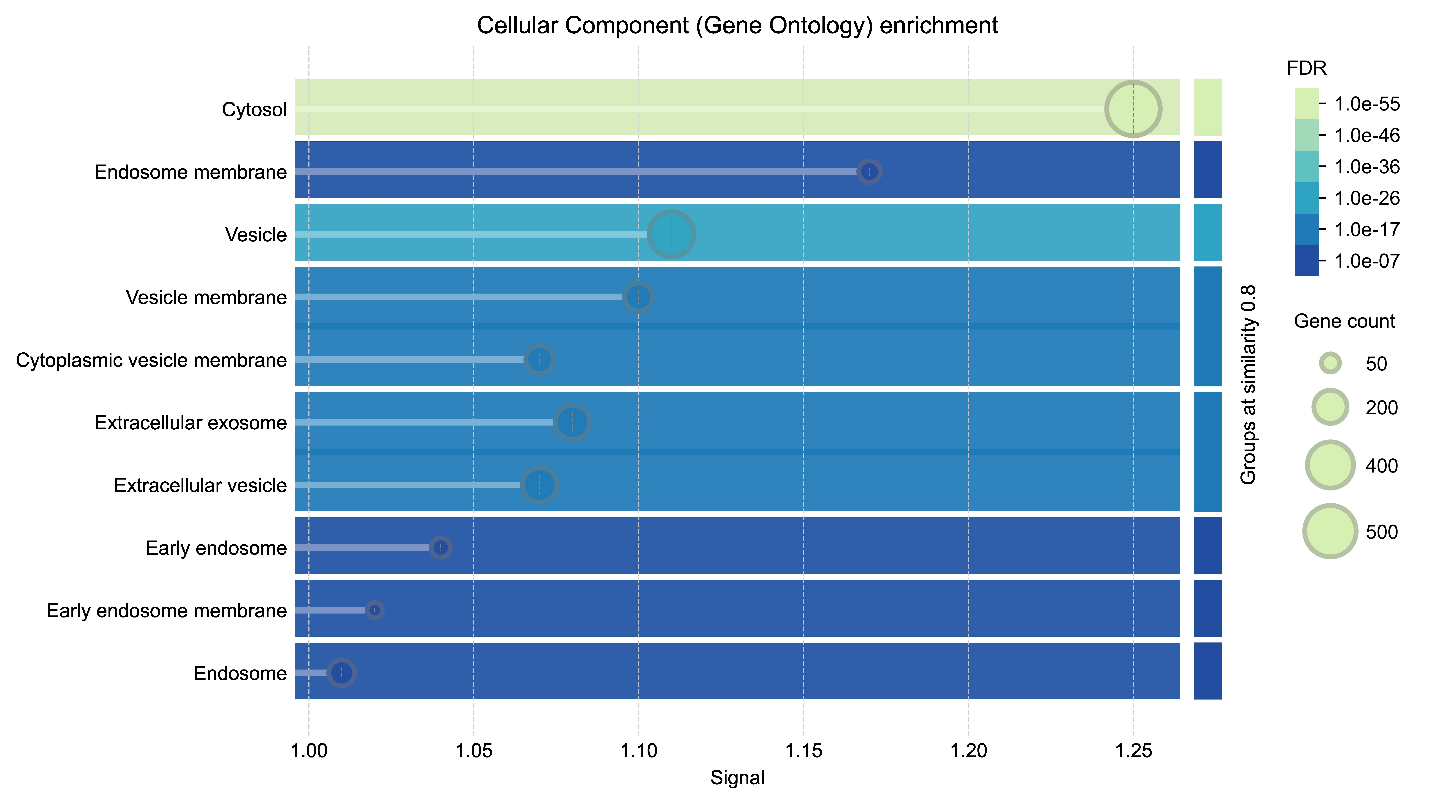


**Figure S3. Cellular Component (Gene Ontology) enrichment of proteins that were present in the 3D-UC, but not in the 3D-TFF/SEC group.** Analysis was performed by STRING.
